# Mutation spectrum of hearing loss patients in Northwest China: Identification of 20 novel variants

**DOI:** 10.1002/mgg3.2434

**Published:** 2024-06-11

**Authors:** Panpan Ma, Bingbo Zhou, Qichao Kang, Xue Chen, Xinyuan Tian, Ling Hui, Shengju Hao, Huiyan Wu, Chuan Zhang

**Affiliations:** ^1^ Medical Genetics Center; Gansu Provincial Clinical Research Center for Birth Defects and Rare Diseases Lanzhou Gansu Provincial Maternity and Child‐Care Hospital Gansu China

## Abstract

**Background:**

Hearing loss (HL) is the most frequent sensory deficit in humans, with strong genetic heterogeneity. The genetic diagnosis of HL is very important to aid treatment decisions and to provide prognostic information and genetic counselling for the patient's family.

**Methods:**

We detected and analysed 362 Chinese non‐syndromic HL patients by screening of variants in 15 hot spot mutations. Subsequently, 40 patients underwent further whole‐exome sequencing (WES) to determine genetic aetiology. The candidate variants were verified using Sanger sequencing. Twenty‐three carrier couples with pathogenic variants or likely pathogenic variants chose to proceed with prenatal diagnosis using Sanger sequencing.

**Results:**

Among the 362 HL patients, 102 were assigned a molecular diagnosis with 52 different variants in 22 deafness genes. A total of 41 (11.33%) cases with the biallelic *GJB2* (OMIM # 220290) gene mutations were detected, and 21 (5.80%) had biallelic *SLC26A4* (OMIM # 605646) mutations. Mitochondrial gene (OMIM # 561000) mutations were detected in seven (1.93%) patients. Twenty of the variants in 15 deafness genes were novel. *SOX10* (OMIM # 602229), *MYO15A* (OMIM # 602666) and *WFS1* (OMIM # 606201) were each detected in two patients. Meanwhile, *OSBPL2* (OMIM # 606731), *RRM2B* (OMIM # 604712), *OTOG* (OMIM # 604487), *STRC* (OMIM # 606440), *PCDH15* (OMIM # 605514), *LOXHD1* (OMIM # 613072), *CDH23* (OMIM # 605516), *TMC1* (OMIM # 606706), *CHD7* (OMIM # 608892), *DIAPH3* (OMIM # 614567), *TBC1D24* (OMIM # 613577), *TIMM8A* (OMIM # 300356), *PTPRQ* (OMIM # 603317), *SALL1* (OMIM # 602218), and *GSDME* (OMIM # 608798) were each detected in one patient. In addition, as regards one couple with a heterozygous variant of *CDH23* and *PCDH15*, respectively, prenatal diagnosis results suggest that the foetus had double heterozygous (DH) variants of *CDH23* and *PCDH15*, which has a high risk to cause ID/F type Usher syndrome.

**Conclusion:**

Our study expanded the spectrum of deafness gene variation, which will contribute to the genetic diagnosis, prenatal diagnosis and the procreation guidance of deaf couple. In addition, the deafness caused by two genes should be paid attention to in the prenatal diagnosis of families with both deaf patients.

## Graphical abstract

We detected and analysed 362 Chinese non‐syndromic HL patients, of whom 102 patients were assigned a molecular diagnosis with 52 different variants in 22 deafness genes. Twenty of the variants in 15 deafness genes were novel. Our study expanded the spectrum of deafness gene variation.

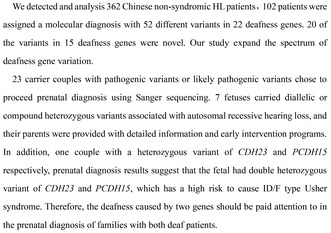

## 1 BACKGROUND

Hearing loss (HL) is one of the most prevalent sensory deficits in humans, with a prevalence of about 2.5/1000 in birth (Abou Tayoun et al., 2016). More than half of HL individuals are affected by genetic factors (Nance et al., 2006). HL includes non‐syndromic HL (NSHL), where the hearing impairment is the only clinical feature, and syndromic with other abnormalities (Zhou et al., 2019). Genetic diagnosis of NSHL is important for the diagnosis, intervention and prevention of inherited HL in patients’ families (Yang et al., 2019). The genetic mode of NSHL is usually subdivided into autosomal recessive (approximately 77%), autosomal dominant (approximately 22%), mitochondrial (approximately 1%) and X/Y‐linked (less) (ACMG, 2002). To date, at least 224 genes have been reported to be associated with HL in humans (https://morl.lab.uiowa.edu/genes‐included‐otoscope‐v9), 66 are autosomal dominant, 117 are autosomal recessive, 21 are autosomal dominant/autosomal recessive, 9 are mitochondrial and 5 are X‐linked (Jin et al., 2022).

The rapid development of next‐generation sequencing (NGS) makes it possible to analyse all genes in one test. Whole‐exome sequencing (WES), a platform of NGS, offers powerful applications for diagnosis as well as for identifying rare variants or new causative genes (Liang et al., 2021).

Here, we recruited 362 Chinese HL families with non‐syndromic hearing loss. Subsequently, we performed DNA microarray test targeting 15 deafness mutations and WES to investigate the contributing genetic factors in patients, which extended the spectrum of deafness‐associated gene variants and provided further guidance for its positive intervention and cure. We provided invasive prenatal diagnosis for 23 carrier couples with pathogenic variants or likely pathogenic variants and helped them deliver healthy babies.

## 2 MATERIALS AND METHODS

### 2.1 Recruitment of patients

Three hundred and sixty‐two patients with non‐syndromic HL were recruited from the outpatient clinic of the Department of Medical Genetics, Gansu Maternity and Child Health Care Hospital (Lanzhou, Gansu, China) in the period 2018–2023. These patients were clinically diagnosed with bilateral sensorineural hearing loss, and audiological tests were performed in the Department of Otolaryngology of the Gansu Maternity and Child Health Care Hospital. Tests included pure‐tone audiometry (PTA, or behavioural audiometry) for patients >4 years old and multiple‐frequency auditory steady‐state evoked response (ASSR) tests for patients ≤4 years old (Guo et al., 2020). All probands were from unrelated Chinese families and some of them were couples trying to have children. The 362 Chinese HL patients included 192 males and 170 females, and they were aged from 3 days to 53 years, and the age of onset ranged from birth to 22 years. This study was undertaken according to the tenets of the Declaration of Helsinki 1975 and its later amendments. The study protocol was approved by the Ethics Committee of the Gansu Provincial Maternity and Child‐Care Hospital (2021GSFY [65]). Written informed consent was obtained from all study participants or their legal guardians.

### 2.2 Genomic DNA extraction

Peripheral blood samples (2–3 mL) were collected from the 362 probands and the parents of 40 patients. Genomic DNA was extracted using a Tiangen DNA extraction kit (Tiangen Biotech, Beijing, China), according to the manufacturer's instructions. DNA purity and concentration were determined by NanoDrop 2000 Spectrophotometer (Thermo Scientifc, USA). The concentration of the qualified sample was 50–250 ng/μL and the absorbance (OD260/OD280) read was 1.8–2.0.

### 2.3 Screening of variants in 15 hot spots mutations

Three hundred and sixty‐two HL probands were detected with 15 genetic deafness detection kits (microarray chip method) (Beijing Boao Biological Co., Ltd., item No. 300068), according to the manufacturer's instructions. The 15 variant hot spots include *GJB2* (NM_004004.6) (c.35delG, c.176del16bp, c.235delC and c.299_300delAT), *GJB3* (OMIM # 603324) (NM_024009.3) (c.538C > T), *SLC26A4* (NM_000441.2) (c.919_2A > G, c.2168A > G, c.1229C > T, c.1975G > C, c.1174A > T, c.1226G > A, c.2027 T > A and c.IVS15 + 5G > A) and *MT‐RNR1* (m.1555A > G, m.1494C > T).

### 2.4 Whole‐exome sequencing and Sanger sequencing

WES was performed by Chigene Co., Ltd. using the GenCap™ Custom Enrichment kit (MyGenostics, Beijing, China). The qualified genomic DNA was randomly fragmented to an average size of 180–280 bp by enzyme digestion. Next, the DNA fragments were end‐repaired and phosphorylated, followed by A‐tailing and ligation at the 3′‐ends with paired end adaptors and index (Illumina). The size distribution and concentration of the libraries were determined by Qubit 3.0 Fluorometer and Agilent 2100 Bioanalyzer system. The library DNA was hybridized with the biotin‐specific probe, and the magnetic beads, which were modified by streptavidin, captured the target gene. Finally, the DNA library was sequenced on NovaSeq 6000 (Illumina, CA, USA). Candidate variants were validated in the proband's parents in each family by Sanger sequencing. Sequencing primers were designed using online Primer Designer Tool Primer 3.0 (http://primer3.ut.ee) and genomic DNA was amplifed using the HotStarTaq Master Mix Kit. The PCR products were bidirectionally sequenced using the BigDye Terminator v3.1 Cycle Sequencing Kit (Applied Biosystems, USA) on an ABI 3500 Dx Genetic Analyzer (Applied Biosystems, USA). Data analysis was conducted using the software SeqMan Pro.

### 2.5 Bioinformatics analysis

Variants were filtered according to their predicted effects and the minor allele frequencies (MAF) <1% in the public database, the Genome Aggregation Database (gnomAD, http://gnomad.broadinstitute.org/), the Exome Aggregation Consortium (ExAC, http://exac.broadinstitute.org) and the 1000 Genomes Browsers (http://browser,1000genomes.org). Novel variants were searched in the Human Gene Mutation Database (HGMD; www.hgmd.cf.ac.uk/), ClinVar database (www.ncbi. nlm.nih.gov/clinvar/) and the Genome Aggregation Database (gnomAD) database. The possible functional role of the novel variants was predicted by PolyPhen‐2 (Polymorphism Phenotyping v2, http://genetics. bwh. harvard. edu /pph2) and Protein Variation Effect Analyzer (PROVEAN) (http://provean.jcvi.org/index.php). The conservativeness of the novel site is evaluated by the software MEGA (Molecular Evolutionary Genetics Analysis). InterVar (http://wintervar. wglab.org/) was used to evaluate the pathogenicity of all variants, according to the standards and guidelines of the American College of Medical Genetics and Genomics (ACMG) (Richards et al., 2015).

## 3 RESULTS

### 3.1 Variant analysis

Pre‐screening of variants in 15 hot spot mutations was performed in all 362 probands, and 69 out of the 362 (19.06%) probands underwent gene diagnosis. The variant of the *GJB2* gene was checked out in 45 (12.43%) patients, including 23 homozygous mutations, 18 compound heterozygous mutations and 4 mono‐heterozygous mutations. The most common pathogenic alleles were c.235delC, c.299‐300delAT and c.176‐c.191del and the allele frequencies were 8.01%, 2.48% and 1.10%, respectively. In addition, 27 (7.46%) probands of *SLC26A4* gene variants were detected, including 12 homozygous mutations, 9 compound heterozygous mutations and 6 mono‐heterozygous mutations. The most common alleles were c.919‐2A > G, c.2168A > G and c.1174A > T, with the allele frequencies of 4.56%, 0.83% and 0.41%, respectively. Moreover, the mitochondrial gene mutations were found in seven (1.93%) patients, including m.1555A > G and m.1095 T > C, with the allele frequencies of 0.83% and 0.14%, respectively (Table 1).

**TABLE 1:** Variants of screening in 15 hot spot mutation among 362 probands with hearing loss.

Gene	Nucleotide change	Amino acid change	Variant type	Allele Counts	Allele frequency in affected subjects (%, in 724 alleles)	Pathogenicity National Center for Biotechnology Information (NCBI)
*GJB2* (NM_004004.6)						
c.235delC		p. L79fs*3	Frameshift	58	8.01	P
c.299_300del		p.H100Rfs*14	Frameshift	18	2.48	P
c.176_c.191del		p.G59Afs*18	Frameshift	8	1.10	P
c.35delG		p.G12Vfs*2	Frameshift	2	0.28	P
*SLC26A4* (NM_000441.2)						
c.919‐2A > G		splicing	Splice site	33	4.56	P
c.2168A > G		p.H723R	Missense	6	0.83	P/LP
c.1174A > T		p.N392Y	Missense	3	0.41	P
c.2027 T > A		p.L676Q	Missense	2	0.28	P/LP
c.1226G > A		p.R409H	Missense	1	0.14	P/LP
c.2162C > T		p.T721M	Missense	1	0.14	P
c.281C > T		p.T94I	Missense	1	0.14	P
c.1707 + 5G > A		splicing	Splice site	1	0.14	p
*MT‐RNR1*						
chrM‐1555A > G				6	0.83	P
chrM‐1494C > T				1	0.14	P

Abbreviations: LP, likely pathogenic; P, pathogenic; VUS, variant of uncertain significance.

Among the remaining 293 HL probands, we recruited 40 HL families for WES to confirm the diagnosis. Thirty‐three out of the 40 (82.50%) patients had variants in known HL genes, which include pathogenic (P), likely pathogenic (LP) or variants of uncertain significance (VUS) in accordance with ACMG, and the remaining seven patients (17.50%) were not identified. Twenty‐one deafness gene variants were detected. The *SLC26A4*, *GJB2* and *GJB3* genes were detected in eight, five and one patients, respectively. Nineteen patients had other known deafness gene variants. *SOX10*, *MYO15A* and *WFS1* were each detected in two patients. Meanwhile *OSBPL2*, *RRM2B*, *OTOG*, *STRC*, *PCDH15*, *LOXHD1*, *CDH23*, *TMC1*, *CHD7*, *DIAPH3*, *TBC1D24*, *TIMM8A*, *PTPRQ*, *SALL1*, and *GSDME* were each detected in one patient (Table 2).

**TABLE 2:** Variant analysis of the HL patients with WES in this study.

ID	Sex	Age	Age of diagnoses	Gene	Inheritance	NM transcript	Nucleotide change	Amino acid change	Variant type	gnomAD allele frequency	Reported or not (PubMed ID)	Disease	Pedigree
1	M	9Y	Birth	*SLC26A4*	AR	NM_000441.2	c.919‐2A > G		Splice site	0.0043	Yes (23151025)	Pendred syndrome	Nuclear family
							c.2000 T > C	p. F667S	Missense	0	Yes (22412181)		
2	M	4.5Y	Birth	*SLC26A4*	AR	NM_000441.2	c.1174A > T	p.N392Y	Missense	0.0024	Yes (21961810)	Pendred syndrome	Nuclear family
							c.203 T > C	p.L68P	Missense	0	Yes (25372295)		
3	M	5 M	Birth	*SLC26A4*	AR	NM_000441.2	c.919‐2A > G		Splice site	0.0043	Yes (23151025)	Pendred syndrome	Nuclear family
							c.1517 T > G	p.L506R	Missense	‐	Yes (23,185,506;23,185,506)		
4	F	6Y	2Y	*SLC26A4*	AR	NM_000441.2	c.1262A > C	p.Q421P	Missense	0.0001	Yes (17718863)	Pendred syndrome	Nuclear family
							c.1991C > T	p.A664V	Missense	‐	Yes (21154317)		
5	M	31Y	Birth	*SLC26A4*	AR	NM_000441.2	c.410C > T	p.S137L	Missense	‐	No	Pendred syndrome	Nuclear family
6	F	29Y	2Y	*SLC26A4*	AR	NM_000441.2	c.919‐2A > G		Splice site	0.0043	Yes (23151025)	Pendred syndrome	Nuclear family
							c.439A > G	p.M147V	Missense	0.00217	Yes (14508505)		
7	F	6Y	2Y	*SLC26A4*	AR	NM_000441.2	c.919‐2A > G		Splice site	0.0043	Yes (23151025)	Pendred syndrome	Nuclear family
							c.227C > T	p.P76L	Missense	‐	Yes (17718863)		
8	M	6Y	Birth	*SLC26A4*	AR	NM_000441.2	c.919‐2A > G		Splice site	0.0043	Yes (23151025)	Pendred syndrome	Nuclear family
							c.1614 + 1G > A		Splice site	0.00008	Yes (11919333)		
9	M	1Y	Birth	*GJB2*	AR	NM_004004.6	c.109G > A	p.V37I	Missense	0.0724	Yes (31160754)	Deafness, autosomal recessive 1A	Nuclear family
							c.235delC	p. L79fs*3	Frameshift	0.0096	Yes (10501520)		
10	M	5 M	Birth	*GJB2*	AR	NM_004004.6	c.109G > A	p.V37I	Missense	0.0724	Yes (31160754)	Deafness, autosomal recessive 1A	Nuclear family
							c.235delC	p. L79fs*3	Frameshift	0.0096	Yes (10501520)		
11	F	24Y	2Y	*GJB2*	AR	NM_004004.6	c.224G > C	p.R75P	Missense	‐	Yes (18924167)	Deafness, autosomal recessive 1A	Nuclear family
12	M	4D	Birth	*GJB2*	AR	NM_004004.6	c.235delC	p. L79fs*3	Frameshift	0.0096	Yes (10501520)	Deafness, autosomal recessive 1A	Nuclear family
							c.35dupG	p.V13Cfs*35	Frameshift	0.0001	Yes (9482292)		
13	F	5 M	Birth	*GJB2*	AR	NM_004004.6	c.235delC	p. L79fs*3	Frameshift	0.0096	Yes (10501520)	Deafness, autosomal recessive 1A	Nuclear family
							c.583A > G	p.M195V	Missense	0.0002	Yes (20497192)		
14	F	36Y	2Y	*GJB3*	AD	NM_024009.3	c.497A > G	p.N166S	Missense	0.0001	Yes (19050930)	Deafness, non‐syndromic, autosomal recessive	Sporadic case
15	F	29Y	5Y	*OSBPL2*	AD	NM_144498.4	c.156_c.157delTC	p.Q53fs*100	Frameshift	0	Yes (25077649)	Deafness, autosomal dominant 67 (DFNA67)	Nuclear family
16	M	8Y	2Y	*RRM2B*	AR	NM_001172477.1	c.636G > C	p.L212F	Missense	‐	No	Progressive external ophthalmoplegia with mitochondrial DNA deletions, autosomal dominant 5 (PEOA5)	Nuclear family
17	M	3D	3D	*OTOG*	AR	NM_001277269.2	c.6322_c.6323insGAGT	p.C2108fs*1	Frameshift	‐	No	Deafness, autosomal recessive 18B (DFNB18B)	Nuclear family
18	F	24Y	Birth	*SOX10*	AD	NM_006941.4	c.332_c.333insGCCTT	p.F111Lfs*37	Frameshift	‐	No	Waardenburg syndrome type 4C	Sporadic case
19	F	7Y	Birth	*SOX10*	AD	NM_006941.4	c.325A > T	p.N109Y	Missense	‐	Yes (27759048)	Waardenburg syndrome	Sporadic case
20	F	2Y	2Y	*STRC*	AR	NM_1 53700.2	loss1 (EXON:1–29) (all)	19,052 bp	missing	‐	No	Deafness, autosomal recessive 16 (DFNB16)	Nuclear family
							c.4561_c.4562insC	p.R1521fs*6	Frameshift	‐	No		
21	M	9Y	3Y	*MYO15A*	AR	NM_016239.4	c.10419_c.10423delCAGCT	p.S3474fs*42	Frameshift	‐	No	Deafness, autosomal recessive 3 (DFNB3)	Nuclear family
							c.10250_c.10252delCCT	p.S3417del	non‐frameshift	0.0005	No		
22	F	21Y	Birth	*MYO15A*	AR	NM_016239.4	c.10419_c.10423delCAGCT	p.S3474fs*42	Frameshift	‐	No	Deafness, autosomal recessive 3 (DFNB3)	Nuclear family
							c.10294_10308delCCTTGCATCCTTGCC	p.P3432_A3436del	non‐frameshift	‐	No		
				*PCDH15*	AR	NM_001354429.2	c.5048_5051dupAGAA	p.N1684fs*11	Frameshift	0.0000166	No	Usher syndrome, type 1D/F digenic	Nuclear family
23	M	28Y	Birth	*LOXHD1*	AR	NM_144612.7	c.6388C > T	p.Q2130X	Nonsense	‐	No	Deafness, autosomal recessive 77	Nuclear family
							exon33‐38del		missing	‐	No		
				*CDH23*	AR	NM_022124.6	c.6693delT	p.F2231fs*3	Frameshift	‐	No	Usher syndrome, type 1D/F digenic	Nuclear family
24	M	29Y	2Y	*TMC1*	AD	NM_138691.3	c.1735G > A	p.A579T	Missense	0.000008	No	Deafness, autosomal dominant 36Deafness, autosomal recessive 7	Nuclear family
25	M	8Y	Birth	*CHD7*	AD	NM_017780.4	c.7252C > T	p.R2418X	Nonsense	‐	Yes (16,155,193;25,525,159)	Charge syndrome	Sporadic case
26	M	23Y		*DIAPH3*	AD	NM_001258370.2	c.2011A > G	p.I671V	Missense	0.000004	No	Auditory neuropathy, autosomal dominant 1	Nuclear family
27	F	6Y	Birth	*TBC1D24*	AR	NM_001199107.2	c.194G > A	p.R65H	Missense	‐	No	Deafness, autosomal recessive 86	Nuclear family
							c.696_697insCTGGTGGA	p.E235Dfs*22	Frameshift	‐	No		
28	M	31Y	3Y	*TIMM8A*	XLR	NM_004085.4	c.1A > G	p.M1V	Missense	‐	No	Mohr–Tranebjaerg syndrome	Nuclear family
29	F	7Y	Birth	*PTPRQ*	AD	NM_001145026.2	c.1135A > G	p.I379V	Missense	0.0002	No	Deafness, autosomal dominant 73	Nuclear family
30	M	6Y	6Y	*WFS1*	AD	NM_006005.3	c.2051C > T	p.A684V	Missense	‐	Yes (11,295,831;21,538,838)	Deafness, autosomal dominant 6/14/38; Wolfram‐like syndrome, autosomal dominant	Sporadic case
31	F	11Y	Birth	*WFS1*	AD	NM_006005.3	c.2051C > T	p.A684V	Missense	‐	Yes (11,295,831;21,538,838)	Deafness, autosomal dominant 6/14/38; Wolfram‐like syndrome, autosomal dominant	Sporadic case
32	F	5Y	Birth	*SALL1*	AD	NM_002968.3	c.3968C > T	p.T1323M	Missense	‐	No	Townes–Brocks syndrome 1	Nuclear family
33	F	27Y	22Y	*GSDME*	AD	NM_004403.3	c.1102C > G	p.Q368E	Missense		Yes (29266521)	Deafness, autosomal dominant 5, DFNA5	Nuclear family

Abbreviations: −, not included in the gnomAD database; F, female; M, male; M, month; Y, years old.

The 33 patients carried 41 different variants, of which 20 were novel, accounting for 48.78% of the total variants (20/41). These 41 variants included 6 different variant types, including 23 missense variants (56.10%, 23/41), 10 frameshift variants (24.39%, 10/41), 2 non‐frameshift variants (4.88%, 2/41), 2 splice site variants (4.88%, 2/41), 2 missing variants (4.88%, 2/41) and 2 nonsense variants (4.88%, 2/41).

We identified 20 novel variants in 15 deafness genes, which were not previously reported in ClinVar or HGMD. According to the ACMG guidelines, 12 variants were classified as pathogenic variant, 4 were likely pathogenic variant and other 4 variants were of uncertain significance (Table 3).

**TABLE 3:** Pathogenicity analysis of novel variants.

Gene	NM transcript	Nucleotide change	Amino acid change	ACMG evidence	Pathogenicity
*SLC26A4*	NM_000441.2	c.410C > T	p.S137L	PM2_Supporting+PM3_Strong+PP3 + PP4	LP
*RRM2B*	NM_001172477.1	c.636G > C	p.L212F	PM2_Supporting+PP3 + PP4	VUS
*OTOG*	NM_001277269.2	c.6322_c.6323insGAGT	p.C2108fs*1	PVS1 + PM2_Supporting+PP4	P
*SOX10*	NM_006941.4	c.332_c.333insGCCTT	p.F111Lfs*37	PVS1 + PM2_Supporting+PP4	P
*STRC*	NM_1 53700.2	loss1 (EXON:1–29) (all)	19,052 bp	PVS1 + PM2_Supporting+PP4	P
*STRC*	NM_1 53700.2	c.4561_c.4562insC	p.R1521fs*6	PVS1 + PM2_Supporting+PP4	P
*MYO15A*	NM_016239.4	c.10419_c.10423delCAGCT	p.S3474fs*42	PVS1 + PM2_Supporting+PP4	P
*MYO15A*	NM_016239.4	c.10250_c.10252delCCT	p.S3417del	PM2_Supporting+PM3 + PM4 + PP4	P
*MYO15A*	NM_016239.4	c.10294_10308delCCTTGCATCCTTGCC	p.P3432_A3436del	PM2_Supporting+PM3 + PM4 + PP4	LP
*PCDH15*	NM_001354429.2	c.5048_5051dupAGAA	p.N1684fs*11	PVS1 + PM2_Supporting+PP4	P
*LOXHD1*	NM_144612.7	c.6388C > T	p.Q2130X	PVS1 + PM2_Supporting+PP4	P
*LOXHD1*	NM_144612.7	exon33‐38del		PVS1 + PM2_Supporting+PP4	P
*CDH23*	NM_022124.6	c.6693delT	p.F2231fs*3	PVS1 + PM2_Supporting+PP4	P
*TMC1*	NM_138691.3	c.1735G > A	p.A579T	PM2_Supporting+PP3 + PP4	VUS
*DIAPH3*	NM_001258370.2	c.2011A > G	p.I671V	PS4 + PM2_Supporting+PP1 + PP3 + PP4	LP
*TBC1D24*	NM_001199107.2	c.194G > A	p.R65H	PM2_Supporting+PM3 + PM5 + PP3 + PP4	LP
*TBC1D24*	NM_001199107.2	c.696_697insCTGGTGGA	p.E235Dfs*22	PVS1 + PM2_Supporting+PP4	P
*TIMM8A*	NM_004085.4	c.1A > G	p.M1V	PVS1 + PM2_Supporting+ PP4	P
*PTPRQ*	NM_001145026.2	c.1135A > G	p.I379V	PM2_Supporting+PP3 + PP4	VUS
*SALL1*	NM_002968.3	c.3968C > T	p.T1323M	PM2_Supporting+PP3 + PP4	VUS

Abbreviations: LP, likely pathogenic; P, pathogenic; VUS, variant of uncertain significance.

### 3.2 Prenatal diagnosis

After receiving the genetic counselling, 23 carrier couples with pathogenic variants or likely pathogenic variants chose to proceed with prenatal diagnosis. As shown in Table 4, seven foetuses harboured neither of the deafness‐causing variants from their parents and 9 foetuses were monoallelic variant carriers. Seven foetuses carried diallelic or compound heterozygous variants associated with autosomal recessive hearing loss, and their parents were provided with detailed information and early intervention programmes.

**TABLE 4:** Prenatal diagnosis analysis of the 23 carrier couples in this study.

Gene	Proband	Foetus	Phenotypes	Pregnancy outcome
*SLC26A4* (NM_000441.2)				
c.919‐2A > G/c.1174A > T		c.919‐2A > G/N	Carrier	Birth, normal
c.919‐2A > G /c.227C > T		c.919‐2A > G/N	Carrier	Birth, normal
c.1262A > C/c.1991C > T		c.1262A > C/c.1991C > T	Patient	Miscarriage
c.2168A > G/c.2168A > G		c.2168A > G/N	Carrier	Birth, normal
c.919‐2A > G/c.919‐2A > G		N/N	Normal	Birth, normal
c.919‐2A > G/c.919‐2A > G		c.919‐2A > G/N	Carrier	Birth, normal
c.919‐2A > G/c.1226G > A		c.1226G > A/N	Carrier	Birth, normal
c.919‐2A > G/c.1707 + 5G > A		c.919‐2A > G/c.1707 + 5G > A	Patient	Miscarriage
*GJB2* (NM_004004.6)				
c.235delC/c.299_300delAT		N/N	Normal	Birth, normal
c.109G > A/c.235delC		c.235delC/N	Carrier	Birth, normal
c.235delC/c.299_300delAT		N/N	Normal	Birth, normal
c.235delC/c.299_300delAT		c.235delC/N	Carrier	Birth, normal
c.299_300del		N/N	Normal	Birth, normal
c.235delC/c.35dupG		c.235delC/N	Carrier	Birth, normal
c.235delC/c.235delC		c.235delC/N	Carrier	Birth, normal
c.235delC/c.235delC		c.235delC/c.235delC	Patient	Miscarriage
c.236delC/c.235delC		N/N	Normal	Birth, normal
c.176_c.191del/c.235delC		c.176_c.191del/ c.235delC	Patient	Miscarriage
*SOX10*(NM_006941.4)				
c.325A > T		N/N	Normal	Birth, normal
c.332_c.333insGCCTT/N		N/N	Normal	Birth, normal
c.332_c.333insGCCTT/N		c.332_c.333insGCCTT/N	Patient	Miscarriage
*GSDME* (NM_004403.3)				
c.1102C > G/N		c.1102C > G/N	Patient	Miscarriage
*CDH23* (NM_022124.6)		c.6693delT	Patient	Miscarriage
*PCDH15* (NM_001354429.2)		c.5048_5051dupAGAA		

Abbreviation: N, no mutation.

One of these couples with HL performed WES at the same time. The wife was detected to carry compound heterozygous variants of the *MYO15A* gene (NM_016239.4): c.10419_10423delCAGCT (p.S3474fs*42) and c.10294_10308delCCTTGCATCCTTGCC (p.P3432_A3436del), which were, respectively, inherited from her father and mother. The gene associated with autosomal recessive deafness‐3 (DFNB3, OMIM # 600316). Meanwhile, the wife carried a heterozygous *PCDH15* (NM_001354429.2) variant, c.5048_5051dupAGAA (p.N1684Kfs*11). The husband was identified by compound heterozygous variants of the *LOXHD1* (NM_144612.7): c.6388C > T (p.Q2130X)/exon 33–38 del, which were, respectively, inherited from his father and mother. The gene associated with autosomal recessive deafness‐77 (DFNB77, OMIM # 613079). In addition, the husband carried a heterozygous *CDH23* (NM_022124.6) variant, c.6693delT (p.F2231Lfs*3). Sanger sequencing was applied to detect amniotic fluid sample of the foetus at the wife's pregnancy of 18 weeks. The foetus was found to have a heterozygous *MYO15A* variant, c.10294_10308delCCTTGCATCCTTGCC (p.P3432_A3436del), and *LOXHD1* variant, exon 33–38 del, which were, respectively, inherited from his mother and father. Unfortunately, the foetus simultaneously detected had a heterozygous *CDH23* variant, c.6693delT (p.F2231Lfs*3), and a heterozygous *PCDH15* variant, c.5048_5051dupAGAA (p.N1684Kfs * 11), which were, respectively, inherited from his father and mother. The two variants of *CDH23* and *PCDH15* have not been reported. *CDH23* is responsible for both DFNB12 (OMIM # 601386) and Usher syndrome 1D (OMIM # 601067) (Bork et al., 2001), whereas *PCDH15* is responsible for both DFNB23 (OMIM # 609533) and Usher syndrome 1F (OMIM # 602083) (Ahmed et al., 2003). Double heterozygous variants of *CDH23* and *PCDH15* have been reported to be a digenic cause of hearing loss (Mutai et al., 2013).

## 4 DISCUSSION

In this study, we performed a molecular diagnosis of 362 unrelated Chinese with HL by screening of variants in 15 hot spot mutations and WES. We determined 52 different variants in 22 deafness genes. We identified 20 novel variants in 15 deafness genes, which were not previously reported in ClinVar or HGMD.

Liu et al. analysed the variant screening results of *GJB2*, *GJB3*, *SLC26A4* and *MT‐RNR1* in 398 symmetrical sensorineural HL patients. Sixty‐nine (17.34%) cases had the biallelic *GJB2* gene variants, and the most common variants were c.235delC, c.109G > A and c.299_300delAT, with allele frequencies of 12.31%, 3.38% and 3.89%, respectively. Sixty‐three (15.83%) cases with biallelic *SLC26A4* variants were detected, and the most common pathogenic alleles were c.919‐2A > G, c.2168A > G and c.1174A > T, with allele frequencies of 9.17%, 2.26% and 0.88%, respectively. Mitochondrial gene variants were detected in nine (2.26%) patients, with five cases of mitochondrial DNA (mtDNA) m.1555A > G variant and four cases of mtDNA m.1095 T > C variant (Liu et al., 2020). Hu et al. recruited 3541 subjects and used multiplex polymerase chain reaction (PCR) combined with next‐generation sequencing to detect 100 hot spot variants in 18 common deafness‐related genes. Thirty‐seven alleles of eight deafness genes were detected. One hundred forty‐five (4.09%) were found to be *GJB2* gene variant carriers, and the hot spot variant was c.235delC (1.54%). Twenty‐three (0.65%) were found to be *GJB3* gene variant carriers. One hundred thirty‐two (3.37%) were found to be *SLC26A4* gene variant carriers, and the hot spot variant was c.919‐2A > G (0.49%). Forty‐four (1.24%) were found to be mitochondrial DNA (mtDNA) variant carriers (Hu et al., 2021).

In our study, *GJB2* mutations were detected in 46 of the 362 probands (12.7%), and the most common variants were c.235delC, with allele frequencies of 8.01%. In addition, 29 probands of *SLC26A4* gene mutations were detected in 362 probands, accounting for 8.01%, and the most common pathogenic alleles were c.919‐2A > G, with allele frequencies of 4.56%. Particularly, we identified a novel variant c.410C > T in *SLC26A4* gene, which was not reported in ClinVar or HGMD previously. According to the ACMG guidelines, this variant was categorized as “likely pathogenic” variant. Furthermore, the mitochondrial gene mutations were found in seven patients, accounting for 1.93%, and *GJB3* was detected in one patient (0.28%). Nineteen patients (5.25%) had other known deafness gene variants. The common variant genes and loci detected in this study were different from those detected in other regions or ethnic groups, which suggested that genetic screening or testing programmes for deafness should be formulated in accordance with the genetic characteristics of the region (Ma et al., 2023).

Cochlear implantation (CI) is an effective form of hearing restoration that improves quality of life and ameliorates the associated economic, social, emotional and neurocognitive consequences of severe‐to‐profound hearing loss. Some patients, however, do not obtain the expected benefit from implantation (Seligman et al., 2022). Variants in deafness genes lead to different pathologies and might result in varied CI outcomes. *GJB2* and *SLC26A4* gene variants are highly prevalent in pre‐lingual HL patients from China. Patients with *GJB2* or *SLC26A4* variants showed better post‐implant auditory performance and speech intelligibility than those without the variants only when implanted before the age of 3.5 years (Wu, Ko, et al., 2015). It has been demonstrated that variants in the *TMPRSS3* (OMIM # 605511) gene were associated with poor CI outcome (Eppsteiner et al., 2012), whereas variants in the *MYO15A*, *TECTA* (OMIM # 602574), and *ACTG1* (OMIM # 102560) genes also showed relatively good auditory performance after implantation (Miyagawa et al., 2013). Moreover, variants in the *PCDH15* and *DFNB59* (OMIM # 610219) genes were associated with poor CI performance (Wu, Lin, et al., 2015). Understanding genetic causes of HL can determine the pattern and course of a patient's HL and may also predict outcomes after CI. The deficiency of this study is that the surgical outcomes of patients were not followed up, and we will make up for this weakness in the future.

The mitochondrial 12S rRNA (ribosomal RNA) gene variant is associated with maternal inheritance, and the application of aminoglycosides resulted in irreversible hearing loss (Chen et al., 2013). The individuals who have 12S rRNA gene variant are sensitive to aminoglycoside drugs, and they can have normal hearing abilities by avoiding the use of aminoglycoside drugs. In the human mtDNA genome, m.1555A > G is the most common mutation in this gene. Hu et al. detected 44 variants in 3541 participants with 12S rRNA variants, and the detection rate of mtDNA 12S rRNA was 1.24% (Hu et al., 2021). We detected 12S rRNA m.1555A > G variant and m.1494C > T in six and one patients, respectively. For these patients, we conducted genetic counselling to avoid deafness induced by the use of aminoglycosides. Therefore, by screening mtDNA 12S rRNA gene, people at high risk of drug‐induced deafness can benefit.

Another important significance of the genetic diagnosis for NSHL is to prevent genetic HL through pregnancy and prenatal genetic diagnosis and counselling. Specific DNA sequencing of the pathogenic or likely pathogenic variant sites was carried out in 23 foetuses. Seven foetuses carried homozygous or compound heterozygous variants associated with autosomal recessive hearing loss, and this diagnosis helped to directly prevent the birth of seven HL children successfully.

To date, 124 genes have been reported to be associated with non‐syndromic HL (http://hereditaryhearingloss.org/). Fifty‐one are autosomal dominant, 77 are autosomal recessive and 5 are X‐linked. The genes have high clinical and genetic heterogeneity. Thus, the prenatal diagnosis should not only focus on identifying monogenic causes of disease, but also on the development of a detection strategy for digenic and oligogenic causes of disease which should be considered in the future. For example, ID/F type Usher syndrome (OMIM # 601067) is caused by double heterozygous variants of *CDH23* and *PCDH15*. In our study, the patient's family would not give birth to patients with HL caused by *LOXHD1* and *MYO15A* according to the rule of autosomal recessive inheritance. However, the couple had the heterozygous variants of *CDH23* and *PCDH15*, respectively, and the foetus had both heterozygous *CDH23* and *PCDH15* variants simultaneously. This combination of double heterozygous variants has been reported (Zheng et al., 2005), and we avoided the birth of a child likely to have hearing loss.

In conclusion, we used the screening of variants in 15 hot spot mutations and WES for genetic diagnosis of 362 NSHL probands. We identified 20 novel variants in 15 deafness genes, which enlarged the variant spectrum of deafness genes in the Han Chinese population. In addition, our study helped to inform the genetic diagnosis and prenatal genetic diagnosis of deaf couple, and added to the theoretical basis for deafness diagnosis, accurate genetic counselling and the procreation guidance.

## AUTHOR CONTRIBUTIONS

PPM and CZ: designed the research; PPM, BBZ, QCK, CX, XYT, LH, HSJ and CZ: analysed the data; PPM and CZ: wrote the paper; and all authors: read, critically revised and approved the final manuscript.

## FUNDING INFORMATION

This work was supported by the Gansu Province Science and Technology Plan Funding Project (22YF7FA094, 20YF8FA093); Lanzhou Science and Technology Plan Project (2021‐1‐182); National Population and Reproductive Health Science Data Center (2005DKA32408); National Science and Technology Resource Sharing Service Platform Project (YCZYPT [2020] 05‐03); Gansu Provincial Clinical Research Center for Birth Defects and Rare Diseases (21JR7RA680); and Gansu Natural Science Foundation (21JR1RA047, 21JR1RA045).

## CONFLICT OF INTEREST STATEMENT

The authors have no conflict of interest to declare.

## ETHICS STATEMENT

The study protocol was approved by the Ethics Committee of the Gansu Provincial Maternity and Child‐Care Hospital (2021GSFY [65]). Written informed consent was obtained from all study participants or their legal guardians.

## LIMITATIONS

Single centre and small sample size.

## Data Availability

The data that support the findings of this study are available on request from the corresponding author. The data are not publicly available due to privacy or ethical restrictions.
